# Chaperoning Roles of Macromolecules Interacting with Proteins *in Vivo*

**DOI:** 10.3390/ijms12031979

**Published:** 2011-03-18

**Authors:** Seong Il Choi, Keo-Heun Lim, Baik L. Seong

**Affiliations:** 1 Translational Research Center for Protein Function Control, Yonsei University, Seoul 120-749, Korea; 2 Department of Biotechnology, College of Bioscience and Biotechnology, Yonsei University, Seoul 120-749, Korea; E-Mail: keoheun@yonsei.ac.kr

**Keywords:** molecular chaperones, macromolecules, hydrophobic interactions, stabilization, aggregation, surface charges, steric hindrance, *de novo* folding

## Abstract

The principles obtained from studies on molecular chaperones have provided explanations for the assisted protein folding *in vivo*. However, the majority of proteins can fold without the assistance of the known molecular chaperones, and little attention has been paid to the potential chaperoning roles of other macromolecules. During protein biogenesis and folding, newly synthesized polypeptide chains interact with a variety of macromolecules, including ribosomes, RNAs, cytoskeleton, lipid bilayer, proteolytic system, *etc*. In general, the hydrophobic interactions between molecular chaperones and their substrates have been widely believed to be mainly responsible for the substrate stabilization against aggregation. Emerging evidence now indicates that other features of macromolecules such as their surface charges, probably resulting in electrostatic repulsions, and steric hindrance, could play a key role in the stabilization of their linked proteins against aggregation. Such stabilizing mechanisms are expected to give new insights into our understanding of the chaperoning functions for *de novo* protein folding. In this review, we will discuss the possible chaperoning roles of these macromolecules in *de novo* folding, based on their charge and steric features.

## 1. Introduction

Proteins frequently encounter misfolding and aggregation during their biogenesis and their life cycles in a cellular environment crowded by macromolecules [1,2], although the amino acid sequences of proteins generally encode the information necessary for their native structures [3]. Moreover, a substantial fraction of proteins have been known to be intrinsically disordered proteins (IDPs) or have intrinsically disordered regions (IDRs) under the physiological conditions [4–6]. The amyloid fibrils and oligomers of many proteins, most of which are IDPs and the proteins with long IDRs (e.g., amyloid-ß, α-synucein, tau, prion protein, and huntingtin) are closely associated with many neurodegenerative diseases [7–9]. Therefore, the understanding of protein aggregation *in vivo* with respect to chaperoning function is of paramount importance in modern biology.

Studies of the representative molecular chaperones such as hsp60 (e.g., GroEL) and hsp70 first introduced the concept of the “assisted” *de novo* folding *in vivo* [10,11]. As a general rule, these chaperones assist protein folding by preventing aggregation (a passive role) in most cases and/or misfolding (an active role, that is, an enhancement of folding rate by inducing global or local conformational changes) in limited cases, via transient binding to the exposed hydrophobic regions of nonnative conformers of substrate proteins [1,2,12–15]. It is evident that the aggregation tendency of proteins strictly depends on their conformational states, thus current studies on the intrinsic or extrinsic factors affecting the protein aggregation have been understood in the context of conformation. Nevertheless, it is the passive role of chaperones, independent of conformational changes, that is mainly responsible for their chaperoning functions. Although the chaperone functions driven by the hydrophobic interaction-mediated substrate recognition and stabilization against aggregation have been the underlying framework for our understanding of the “assisted” protein folding *in vivo*, it still remains unknown what features of the chaperones are important for their substrate stabilization. Recent biochemical and genetic studies have shown that the majority of newly synthesized proteins can fold without assistance of the known chaperones [16–21]. The folding of about ∼3% of *E. coli* proteins was predicted to be significantly dependent on GroEL [19]. Consistently, GroEL depletion using the tightly controlled system was reported to have little effect on *de novo* folding of the majority of *E. coli* proteins [20]. It should be also noted that the GroEL gene is absent or non-essential in some eubacteria [21]. In addition to *de novo* folding, chaperones play crucial roles in the aggregation inhibition of damaged proteins, disaggregation, protein translocation, proteolysis, protein maturation, and signal transduction [11,22–26].

Here we suggest that there might be other chaperone types and mechanisms operating in *de novo* folding *in vivo*, basically distinct from the classical chaperones and their known mechanisms. So far, the chaperoning functions *in vivo* have been understood mainly in terms of conformational changes and intermolecular hydrophobic interactions. Both factors can be described by a bimolecular interaction system. Protein aggregation is a multimolecular and even specific process [27,28]. Especially in multimolecular assembly processes, the intrinsic properties of macromolecules, such as their surface charges and steric hindrance by excluded volume repulsion, might play an important role in stabilizing the interacting aggregation-prone polypeptides. Indeed, newly synthesized polypeptides interact directly or indirectly with a variety of macromolecules *in vivo*. Based on the above mentioned charge and steric factors, we will discuss the potential chaperoning roles of interacting macromolecules in *de novo* protein folding *in vivo*.

## 2. Macromolecule-Mediated Chaperone Type Based on Their Surface Charges and Steric Hindrance

### 2.1. Accumulating Evidence for Charge and Steric Hindrance as Important Stabilizing Factors

Hydrophobic interactions have long been widely accepted to be major driving forces for protein folding and protein aggregation in the aqueous environment [29,30]. Thus, the direct masking of the exposed hydrophobic regions by intermolecular hydrophobic interactions has been widely believed to be a major factor responsible for stabilizing aggregation-prone polypeptides. However, there are other well-known stabilizing factors, distinct from the hydrophobic masking. First, the charge effects on protein solubility are obvious, as evidenced by the following observations. Charged residues interrupt continuous hydrophobic residues in protein sequences as “structural gatekeepers” [31]. A close correlation of net charge with protein solubility has been well documented [32–36]. Strikingly, relatively high net charge observed in IDPs compared to the classical globular proteins serves to maintain their solubility under the physiological conditions [32,34], highlighting the charge effect on protein solubility. Moreover, many IDPs can act as chaperones, and the unstructured regions of chaperones are important for their actions [26,37]. Mechanistically, these unstructured regions were suggested to exert a solubilizing effect on nonnative substrates due to their highly hydrophilic property and entropic exclusion of other molecules [37]. The fusion of a small charged tag to the *N*- or *C*-terminus can improve the solubility of the tagged proteins in some cases [38–40]. Mechanistically, intermolecular electrostatic repulsions by charged residues are widely believed to be important for protein solubility [33,35,36]. Second, large polymers such as glycan and PEG were suggested to inhibit the aggregation of their linked proteins due to their steric hindrance [41,42]. Given that protein aggregation is a self assembly process as mentioned above, the inhibition of protein aggregation by the steric hindrance of the bound bulky macromolecules appears to be highly reasonable. In the colloidal aggregation that has long been studied, both electrostatic repulsions of surface charges and steric hindrance of the polymers absorbed on colloidal surfaces have been known to be major factors for stabilizing colloids against aggregation [43,44]. The supposed action mechanisms of aggregation inhibition by surface charges and steric hindrance are different from those by conformational changes and hydrophobic masking, which will be discussed in more detail.

### *2.2.* N*-Terminal Domains as Solubility Enhancers for Their Linked Domains*

Empirically, the linkage of aggregation-prone proteins to soluble carriers has been known to be an effective way to stabilize proteins against aggregation, although the molecular mechanisms remain unknown [45]. Indeed, the fusion of soluble protein to the *N*-terminus of aggregation-prone protein is currently the most efficient tool to overcome the aggregation of heterologous proteins expressed in the *E. coli* cytoplasm [46,47], whereas the coexpression of chaperones has been successful for soluble expression of target proteins only in limited cases [48]. Despite the popularity of this fusion technology, artificial “tagging” has been considered to be biologically irrelevant. It could be argued, however, that the fusion proteins mimic multidomain proteins in which the *N*-terminal domain acts as a solubility enhancer for the downstream domains, prompting us to speculate that this chaperoning type in an artificial construct could be employed in *de novo* folding of native multidomain proteins *in vivo*. Indeed, the *N*-terminal domains of native multidomain proteins have the ability to solubilize their *C*-terminally fused various heterologous proteins *in vivo*, which suggests that the native *N*-terminal domains have the potential to assist *de novo* folding of their authentic downstream domains *in vivo* by acting as solubility enhancers [49]. Traditionally, multidomain proteins, because of their high propensity to aggregation, were thought to require assistance of chaperones [2,50]. In contrast, these results provided a possible chaperoning role of the cotranslationally or independently folded domains for their linked domains, contributing to the autonomous folding of multidomain proteins *in vivo*.

**Figure 1.**
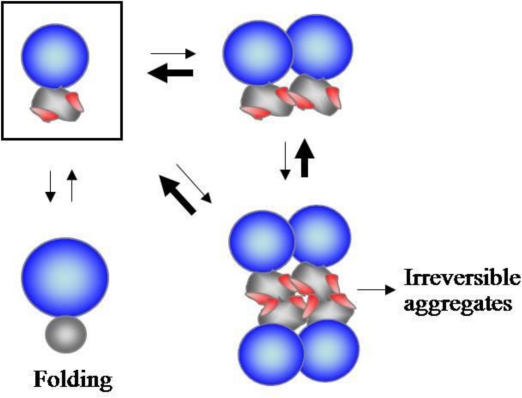
A model for how *N*-terminal domains solubilize their linked domains. The blue, gray, and wrinkled spheres represent the folded *N*- and *C*-terminal domains, and incompletely folded *C*-terminal domains, respectively. The red spots on wrinkled spheres indicate the exposed regions involved in the intermolecular interactions. Thick arrows represent the shift from the oligomeric state to the monomeric state (boxed) of proteins driven by the electrostatic repulsions and steric hindrance of folded *N*-terminal domains. (Reproduced from Reference [49]).

We also showed that the solubilizing ability of the *N*-terminal domains, including the solubility enhancers used in the fusion protein technology, strongly correlates with their net charge and size. Based on these observations, we proposed a model of how folded *N*-terminal domains could solubilize their linked domains, as illustrated in Figure 1. The electrostatic repulsions and steric hindrance of folded *N*-terminal domains could prevent the oligomerization driven by the *C*-terminal aggregation-prone domains, shifting the oligomeric states toward the monomeric states, and thus keeping the *C*-terminal domains in a folding-competent state. In particular, the folded domains could exert a chaperoning activity on their linked domains even without direct contact to the aggregation-prone regions and even without native interdomain interactions, potentially enabling this chaperoning type to be applied to a broad range of multidomain proteins. This model well explains why the linkage of aggregation-prone proteins to large soluble carriers generally improves protein solubility.

### 2.3. Substrate Stabilizing Factors of DnaK

Historically, the hydrophobic interaction-mediated chaperoning mechanism originated from Pelham’s speculations on the action mechanism of hsp70 [11]; “*during heat shock, proteins become partially denatured, exposing hydrophobic regions which then interact to form insoluble aggregates. By binding tightly to hydrophobic surfaces, hsp70 limits such interactions and promote disaggregation*.” Consistent with this prediction, hsp70 as well as other chaperones generally recognize their substrates largely via hydrophobic interactions [2,51,52]. DnaK (an *E. coli* hsp70 homolog) recognizes short linear peptides with 2–4 contiguous hydrophobic residues flanked by basic residues (e.g., NRLLLTG) [51,53]. Notably, DnaK binds a tiny fraction of hydrophobic regions of its substrates. In contrast, BiP, an hsp70 homolog in the endoplasmic reticulum (ER), can recognize the hydrophilic peptides without hydrophobic residues [54]. Even hsp60 and TF can recognize their substrates by electrostatic interactions [55,56]. The major chaperones in ER, calnexin and calrecticulin, recognize their substrates by binding to the glycan moiety of substrates [57]. These observations raise fundamental questions as to whether the intermolecular hydrophobic interaction is a major substrate-stabilizing factor of the chaperones or a tool for the recognition of nonnative substrates or other regulatory functions such as protein translocation and quality control.

**Figure 2.**
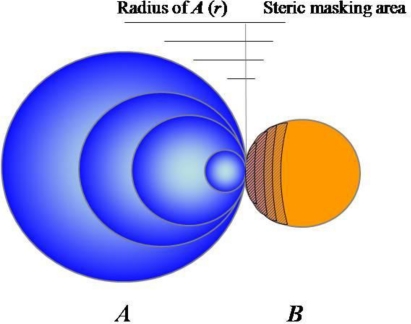
A schematic illustration of substrate-stabilizing factors of macromolecules and their correlation with the size of the macromolecule. Here, an example of a soluble macromolecule, DnaK, with varying radius *r* and constant surface charge density and its bound aggregation-prone protein are represented as sphere ***A*** and ***B***, respectively. The potential factors of sphere ***A*** such as electrostatic repulsions, steric hindrance, and hydrophobic shielding are considered as a function of the radius *r* of sphere ***A***. The hatched area represents the surfaces inaccessible to other ***B*** by the steric masking of the corresponding ***A***. (Adapted from Reference [58]).

Would DnaK have an intrinsic and additional stabilizing ability to substrate proteins irrespective of its hydrophobic interactions with substrates? To address this issue, the aggregation-prone proteins were fused to the *C*-termini of DnaK and its variants with a point mutation in the residue critical for the substrate recognition or deletion of the *C*-terminal substrate-binding domain [58]. Here, the assumption was that the covalent linkage can mimic the noncovalent association between DnaK and its substrate. There was no significant difference in the *cis*-acting solubilizing ability between DnaK and its variants *in vivo*, indicating that DnaK has an intrinsic substrate-stabilizing ability, irrespective of its hydrophobic masking by direct contacts. Based on these results, we proposed a simplified model to explain what factors of macromolecules, including DnaK, can stabilize their linked substrates (Figure 2). In this oversimplified model, a soluble macromolecule (sphere ***A***) with varying radius (*r*) but constant surface charge density is associated with an aggregation-prone protein (sphere ***B***) via limited hydrophobic contact. As radius (*r*) of sphere ***A*** increases, its surface net charge (related to electrostatic repulsion) and excluded volume (related to steric hindrance) are proportional to *r*^2^ and *r*^3^, respectively, whereas the hydrophobic contact area is constant. This suggests that both surface charges and steric hindrance of large soluble macromolecules, including chaperones, would provide dominant stabilizing factors as relative to hydrophobic interactions. An important implication of this model is that soluble macromolecules could have the intrinsic ability to stabilize their linked aggregation-prone polypeptide chains against aggregation, independent of the nature of linkage between them.

The hsp70 can actively unfold its substrates by inducing local conformational changes through ATP hydrolysis. Seemingly, our model does not include this mechanism. An entropic pulling mechanism was proposed to underlie the functions of hsp70 as diverse as inhibition of aggregation, unfolding, disaggregation, and membrane translocation [23]. In this model, the hsp70 has the tendency to move away from protein aggregates or membrane surfaces for more freedom, generating an entropic pulling force; in the closer proximity to the surfaces, hsp70 has less freedom due to its excluded volume. Interestingly, the entropic pulling force and steric hindrance in our model come from a common origin or the excluded volume of hsp70.

### 2.4. RNA-Mediated Chaperone Type

Nascent polypeptides emerging from ribosomes, prior to the formation of stable structure, were thought to be highly aggregation-prone due to the increased effective concentration by close proximity of identical chains on the polysomes and the macromolecular crowding effect in the cytosol [2,59,60]. The aggregation problems of nascent chains on ribosomes have provided a rationale for the existence of the ribosome-associated chaperones such as trigger factor [61,62]. However, their contribution to *de novo* folding on ribosomes still remains unknown [62]. Rather, these factors were recently reported to play an important role in ribosome assembly [56,63,64]. Therefore, it still remains an outstanding issue how the aggregation of the nascent chains on the ribosomes is prevented *in vivo*.

In terms of this issue, the effects of the physical linkage to ribosome on the aggregation behavior of the nascent chains have not been given due consideration. The ribosome is a gigantic RNP complex (its size is approximately 2.6 × 10^6^ dalton in *E. coli*) in which RNAs (polyanionic macromolecules) provide basic structural frames. From the viewpoint of charge and steric factors, in Figure 2, ribosome and large RNAs are ideal chaperoning macromolecules, implying that the RNA-mediated chaperoning functions might be ubiquitous *in vivo*. Indeed, the ribosome, its 23S rRNA, and the V domain of 23S rRNA have been known to function as molecular chaperones *in vitro* [65,66]. Here, the substrate recognition was mediated by the peptidyl transferase center (PTC). However, during protein synthesis, the PTC is expected to be difficult for the nascent chains on the exit sites of ribosomes to physically access.

**Figure 3.**
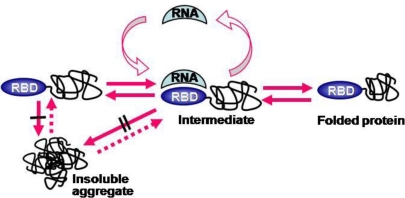
A model for RNA binding-mediated protein folding. Both the folded RNA-binding domain (RBD) at the *N*-terminal position and bound RNA prevent inter-molecular interactions among folding intermediates, leading to soluble expression and favoring kinetic network into productive folding. The number of black bars (| and ||) represents the extent of aggregation inhibition. (Reproduced from Reference [67]).

We previously showed that large RNAs can increase the solubility and folding of their linked proteins, as shown in Figure 3 [67]. When an RNA-binding domain (RBD) is used as a soluble carrier, the RNA binding to RBD (RNP complex) further promoted the solubility of whole proteins and the proper folding of *C*-terminal proteins. The similarity between the RNP-linked aggregation-prone proteins and the ribosome-linked nascent chains made us speculate that ribosomes might contribute to the solubility enhancement of their linked nascent chains in a *cis*-acting manner. Indeed, the ribosome displays technology has been known to be very effective for promoting the solubility and folding of highly aggregation-prone proteins [68,69]. By combining these observations with the model in Figure 2, we proposed that the aggregation-prone nascent chains on ribosomes might gain aggregation-resistance due to the gigantic size and overall negative surface charges of ribosomes [70]. This *cis*-acting chaperoning role of ribosomes has the potential to alleviate the aggregation problems of nascent chains on them. In addition, the three-dimensional organization of bacterial polysomes showed that the polypeptide exit sites are positioned to maximize the distance between them for reducing intermolecular interactions of nascent chains [71]. Thus, the aggregation problems of nascent chains on ribosomes should be understood in the ribosome linkage context.

## 3. Perspectives

Here we discussed that macromolecule-mediated chaperoning types and mechanisms might exist in *de novo* protein folding inside cells. In particular, two intrinsic properties, charge and steric hindrance, of soluble macromolecules were emphasized as to having an important role in stabilizing their linked proteins against aggregation. Given that a variety of soluble macromolecules are linked to aggregation-prone polypeptides *in vivo*, the chaperoning roles of these macromolecules presented here could give new insights into *de novo* protein folding *in vivo*.

The above chaperoning types and mechanisms might be applied to multimolecular assemblies such as amorphous aggregation, ordered aggregation, nonnative or native oligomerization. For example, the members of the hsp70 family are involved in diverse multimolecular associations such as amorphous aggregation, ordered aggregation, oligomerization, and the assembly/disassembly of clathrin and virus particles [11,72,73]. The chaperoning mechanisms mediated by charge and steric factors as discussed in this review are not mutually exclusive with those exerted by conformational changes and hydrophobic interactions. Thus, the idea of a combination of these factors would advance our understanding of the roles of interacting macromolecules in the multimolecular assembly processes.
